# Albumin dialysis improves hepatic encephalopathy and decreases circulating phenolic aromatic amino acids in patients with alcoholic hepatitis and severe liver failure

**DOI:** 10.1186/cc7697

**Published:** 2009-01-28

**Authors:** Albert Parés, Ramón Deulofeu, Laura Cisneros, Angels Escorsell, Joan Manuel Salmerón, Joan Caballería, Antoni Mas

**Affiliations:** 1 Liver Unit, Digestive Diseases Institute. Hospital Clínic, Centro de Investigaciones Biomédicas en Red de Enfermedades Hepáticas y Digestivas (CIBERehd), Institut d'Investigacions Biomèdiques August Pi i Sunyer (IDIBAPS), C/. Villarroel 170, Barcelona, 08036, Spain

## Abstract

**Introduction:**

The aim of this study was to assess the effects of albumin dialysis on hepatic encephalopathy and circulating levels of amino acids in severe alcoholic hepatitis.

**Methods:**

The study was carried out in nine patients with severe alcoholic hepatitis and four with primary biliary cirrhosis treated with the molecular adsorbent recirculating system. Besides standard liver function tests, circulating levels of ammonia, total, branched chain and aromatic amino acids, the presence and severity of hepatic encephalopathy, and number connection test were measured before and after each treatment.

**Results:**

There were eight episodes of encephalopathy in patients with alcoholic hepatitis. Albumin dialysis was associated with significant improvement in encephalopathy (p = 0.02), and a decrease in total amino acid levels (2490 ± 152 μM to 2229 ± 114 μM, p < 0.001). Moreover, the Fischer's ratio, which was significantly lower in patients with alcoholic hepatitis (1.32 ± 0.08) than in controls (3.20 ± 0.16), increased by 17% after albumin dialysis (p < 0.02) because of a significant decrease in phenolic aromatic amino acids (193 ± 17 μM to 165 ± 9 μM, p = 0.04). No differences were observed in circulating ammonia. Changes in phenolic aromatic amino acids and the Fischer's ratio were more prominent in patients with encephalopathy and higher bilirubin removal. Albumin dialysis did not significantly affect the amino acid profile in the controls.

**Conclusions:**

Albumin dialysis results in a significant decrease in circulating phenolic aromatic amino acids and improvement of hepatic encephalopathy in patients with severe liver failure.

## Introduction

Hepatic encephalopathy frequently complicates both acute liver failure and end-stage chronic liver disease [1-3]. The pathophysiological mechanisms of hepatic encephalopathy are poorly understood, although alterations in both the cerebral microcirculation and neuronal function associated with an excessive amount of toxic circulating substances not metabolised by the liver have been implicated [4]. High ammonia levels [5] and an imbalance between aromatic and branched amino acids are some of the mechanisms involved [6-9]. Thus, high levels of phenolic aromatic amino acids (tyrosine and phenylalanine) have been associated with the development of encephalopathy in patients with liver diseases. Moreover, the ratio between branched chain amino acids and phenolic aromatic amino acids has been suggested to correlate with the degree of encephalopathy [8].

A new 'detoxifying' method, based on albumin dialysis or Molecular Adsorbent Recirculating System (MARS), has been launched in the past decade [10,11]. This procedure effectively decreases hepatic encephalopathy in patients with liver failure [12-14]. However, the mechanisms responsible for this improvement, and their relationship with circulating levels of amino acids, are poorly identified. Therefore, the aim of the current study was to assess the effect of albumin dialysis on hepatic encephalopathy and on the circulating levels of amino acids and ammonia in patients with severe liver failure.

## Materials and methods

The study was carried out in a series of nine patients with biopsy proven severe alcoholic hepatitis (six with cirrhosis) defined by a total serum bilirubin level above 10 mg/dl and a prothrombin index lower than 50%, and in a control group of four patients with primary biliary cirrhosis and resistant pruritus. The results of the effect of albumin dialysis on pruritus in these latter patients have already been published [15]. These four patients had circulating albumin, bilirubin and prothrombin index within the normal range. Patients with alcoholic hepatitis were not treated with corticosteroids before and during albumin dialysis and they were under standard medical therapy. No specific treatments for hepatic encephalopathy were used during albumin dialysis. The study protocol conforms to the ethical guidelines of the Declaration of Helsinki and was approved by the Hospital Clínic, Barcelona ethics committee. Patients were included after giving informed written consent (next-of-kin assent in encephalopathic patients).

The study evaluates 23 seven-hour sessions of albumin dialysis performed in the patients with severe alcoholic hepatitis and eight seven-hour sessions performed in the control patients with resistant pruritus. All the patients were haemodynamically stable, did not require additional respiratory or circulatory support and had no evidence of severe infection or multiorgan failure. The following parameters were assessed before and after each treatment: standard liver function tests, arterial ammonia levels, and the concentration of total amino acids, branched amino acids, phenolic aromatic amino acids and tryptophan by high-performance liquid chromatography. Briefly, the blood samples were immediately transferred to chilled heparinised tubes, placed on ice and within five minutes were centrifuged at 3000 rpm for 15 minutes at 4°C and then kept frozen and stored at -80°C until analysis. Amino acids were separated by reversed phase high-performance liquid chromatography after phenyl-iso-thiocyanate derivatisation, to obtain a phenyl-thio-carbamyl derivative (Pico-Tag method, Waters, Milford, MA, USA) and subsequent ultraviolet detection in an automatic high-performance liquid chromatography system and data processing software. The internal standard was methionine sulfone. The coefficient of variation was less than 5% for all amino acid measurements.

### Hepatic encephalopathy

Patients were evaluated by the same physician (LC) before and after each dialysis session for the presence and severity of hepatic encephalopathy according to the West Haven Criteria for semiquantitative grading of mental state [16]. The number connection test was assessed as a measure of cognitive motor abilities [17,18]. The patient has to order numbers printed on a piece of paper consecutively from 1 to 25, as quickly as possible. Errors are not enumerated, but patients are instructed to return to the preceding correct number and then carry on. The test score is the time the patient needs to perform the test, including the time needed to correct the errors.

### Albumin dialysis treatment

Extracorporeal albumin dialysis was performed with MARS (Gambro Lundia AB, Lund, Sweden). It is an extracorporeal liver support device, using a hollow-fibre dialysis column in which the blood of the patient is dialysed across an albumin-coated membrane, while at the same time maintaining a constant flow of albumin-rich dialysate in the extracapillary compartment. Substances with a molecular weight greater than 50 KD are not removed from the plasma by the system [19]. The albumin-enriched dialysate, containing 10 to 20% human serum albumin, is recirculated and its binding sites regenerated by online perfusion through a charcoal column and an anion-exchanger column, and simultaneously dialysated against a bicarbonate-buffered solution using a standard dialysis machine. Therefore, the albumin-bound toxins can be removed through MARS and the water-soluble toxins through haemodialysis. In the current study each dialysis session was performed using a double-lumen catheter in a femoral vein for blood access, and the albumin-enriched dialysate contained 600 ml of 20% human serum albumin. The extracorporeal blood flow and MARS flow were maintained at 250 mL/minute and the dialysate was maintained at body temperature to avoid cooling of the patient. A continuous infusion of heparin at a dose of 1500 to 4000 IU/hour was used as an anticoagulant when necessary.

### Statistical analyses

Data are expressed as mean ± standard error of the mean (SEM). The unpaired students' t-test was used to compare data from patients and controls, and paired students' t test or Wilcoxon test were used when appropriate to analyse differences before and after the procedure. A probability level of 5% was regarded as statistically significant.

## Results

Baseline clinical and analytical abnormalities for patients with alcoholic hepatitis and primary biliary cirrhosis with pruritus are shown in Table 1. Patients with alcoholic hepatitis had severe liver function impairment as compared with patients with pruritus. Thus, all the patients with alcoholic hepatitis had a Maddrey's discriminant function higher than 32 (mean ± SEM: 74.5 ± 6.0) and the Model for End-stage Liver Disease score was 24.3 ± 4.1. Hepatic encephalopathy was detected in five patients (grade I in four patients and grade II in one patient) and the number connection test was also significantly higher in these patients with alcoholic hepatitis. Additionally, seven patients with alcoholic hepatitis had ascites. Total amino acid, as well as phenolic aromatic amino acid, concentrations were significantly higher in patients with alcoholic hepatitis than in patients with pruritus, as were tryptophan levels. No significant differences were found in baseline branched amino acid levels between the two groups of patients but the Fischer ratio was significantly lower in patients with alcoholic hepatitis. Circulating ammonia levels were higher in patients with alcoholic hepatitis as compared with patients with resistant pruritus. Thus, ammonium was elevated in four patients with alcoholic hepatitis and normal in the cases with pruritus. Ammonium was above the normal values (9 to 33 μM/L) before treatment in 12 of the 23 sessions (52.2%) performed in patients with alcoholic hepatitis.

**Table 1:** Clinical and biochemical data of patients with alcoholic hepatitis and patients with resistant pruritus before albumin dialysis

	Patients with severe alcoholic hepatitis n = 9	Patients with resistant pruritus n = 4	p =
Age (years)	48.2 ± 2.0	52.0 ± 4.2	n.s.
Male/Female	6/3	1/3	
Ascites	7	0	
Encephalopathy	5	0	
NCT (minute)	3.68 ± 0.97 (5)	1.01 ± 0.09	0.05
Bilirubin (mg/dl)	22.8 ± 2.8	1.08 ± 0.05	0.005
AST (u/L)	96 ± 11	52 ± 7	0.031
ALT (u/L)	48 ± 4	93 ± 40	n.s.
AP (u/L)	398 ± 53	999 ± 544	0.031
γGT (u/L)	202 ± 65	449 ± 426	n.s.
Albumin (g/L)	24.4 ± 1.03	36.5 ± 1.2	0.005
Prothrombin index (%)	33.2 ± 2.8	91.5 ± 8.5	0.005
Creatinine	1.7 ± 0.8	0.8 ± 0.1	n.s.
Serum Na	130 ± 3	138 ± 2	0.01
Serum K	3.5 ± 0.1	3.9 ± 0.2	0.03
Haemoglobin (g/L)	10.5 ± 0.5	11.8 ± 1.1	n.s.
Leucocytes (cell/×10^9^)	12.9 ± 2.4	8.6 ± 3.7	n.s.
Platelets (cell/×10^9^)	105 ± 14	264 ± 51	0.005
			
Total amino acids (μM/L)	2692 ± 274	1850 ± 93	0.01
Branched chain AA (μM/L)	253 ± 37	331 ± 20	n.s.
Phenolic aromatic AA (μM/L)	186 ± 26	111 ± 8	0.04
Fischer index	1.32 ± 0.15	3.01 ± 0.12	0.005
Tryptophan (μM/L)	28.2 ± 2.8	39.7 ± 2.7	0.02
			
Ammonium (μM/L)	45.2 ± 11.6	23.2 ± 4.7	n.s.

AA = amino acids; ALT = alanine aminotransferase AP = alkaline phosphatase; AST = aspartate aminotransferase; γGT = gamma-glytamyl transpeptidase; K = potassium; Na = sodium; n.s. = not significant; NCT = number connection test.

Eight episodes of encephalopathy were recorded before treatment in the nine patients with alcoholic hepatitis. Albumin dialysis was associated with a significant improvement in the degree of hepatic encephalopathy (p = 0.02), and no encephalopathy was present after treatment in five of the eight episodes. The number connection test decreased in 11 of the 15 cases with alcoholic hepatitis with assessments before and after treatment. No significant changes were observed in the test before and after treatment in patients with pruritus. Marked attenuations in circulating bilirubin and a significant improvement in albumin concentration were also observed after dialysis in these patients, along with clear recoveries in creatinine and electrolyte concentrations. Haemoglobin and platelets decreased significantly after treatment. No significant changes were observed in patients with pruritus except for a significant decrease in the platelet count (Table 2). The improvement in hepatic encephalopathy was not associated with changes in creatinine, sodium or potassium levels after treatment.

**Table 2:** Clinical and biochemical data of patients with alcoholic hepatitis and patients with resistant pruritus before and after the seven-hour sessions of albumin dialysis

	Patients with alcoholic hepatitis n dialysis = 23			Patients with resistant pruritus n dialysis = 8		
	Before	After	p	Before	After	p

Encephalopathy (score)	0.36 ± 0.11	0.16 ± 0.09	0.02	0	0	n.s.
NCT (minute)	3.28 ± 0.43*	2.8 ± 0.31	n.s.	1.17 ± 0.11	1.03 ± 0.08	n.s.
Bilirubin (mg/dl)	20.6 ± 1.5	16.3 ± 1.5	0.001	1.01 ± 0.1	1.2 ± 0.1	n.s.
Albumin (g/L)	24.2 ± 0.6	25.5 ± 0.7	0.03	37.4 ± 0.7	38.2 ± 1.4	n.s.
Prothrombin index (%)	34.4 ± 1.9	30.3 ± 2.2	0.03	92.0 ± 4.8	84.1 ± 5.9	n.s.
Creatinine	1.3 ± 0.3	0.7 ± 0.1	0.008	0.85 ± 0.05	0.78 ± 0.12	n.s.
Serum Na	134 ± 1.0	138 ± 0.6	0.0003	138 ± 0.4	138 ± 0.4	n.s.
Serum K	3.7 ± 0.1	4.0 ± 0.1	0.05	4.1 ± 0.1	4.5 ± 0.2	0.04
Haemoglobin (g/L)	10.0 ± 0.4	9.4 ± 0.4	0.002	11.8 ± 0.4	11.4 ± 0.6	n.s.
Leucocytes (cell/×10^9^)	11.9 ± 1.4	11.1 ± 1.4	0.06	8.2 ± 1.2	7.3 ± 0.6	n.s.
Platelets (cell/×10^9^)	78.6 ± 7.0	56.0 ± 6.0	0.0000	234 ± 19	187 ± 19	0.01
						
Total amino acids (μM/L)	2491 ± 152	2229 ± 114	0.01	2080 ± 159	2354 ± 158	n.s.
Branched AA (μM/L)	243 ± 18	233 ± 12	n.s.	399 ± 44	443 ± 47	n.s.
Phenolic aromatic AA (μM/L)	193 ± 17	165 ± 10	0.04	123 ± 10	145 ± 11	0.05
Fischer index	1.32 ± 0.08	1.47 ± 0.05	0.01	3.20 ± 0.16	3.08 ± 0.24	n.s.
Tryptophan (μM/L)	28.4 ± 1.9	26.0 ± 1.8	n.s.	45.0 ± 2.6	42.9 ± 4.8	n.s.
						
Ammonium (μM/L)	46.2 ± 6.6	41.1 ± 5.5	n.s.	22.2 ± 4.1	21.5 ± 3.2	n.s.

*Assessed in 15 of the 23 treatments.

AA = amino acids; K = potassium; Na = sodium; n.s. = not significant; NCT = number connection test.

**Figure 1:**
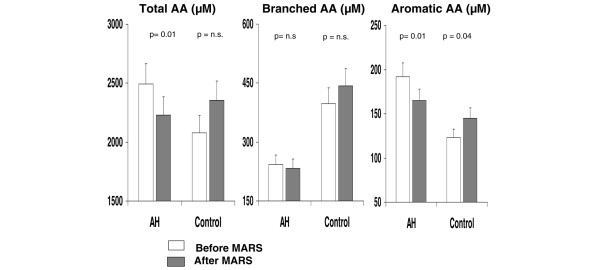
**Circulating levels of total, branched and aromatic amino acids**. Results are shown in patients with alcoholic hepatitis (AH) and pruritus (control) before (empty bars) and after (shaded bars) albumin dialysis. AA = amino acids; MARS = Molecular Adsorbent Recirculating System; n.s. = not significant.

**Figure 2:**
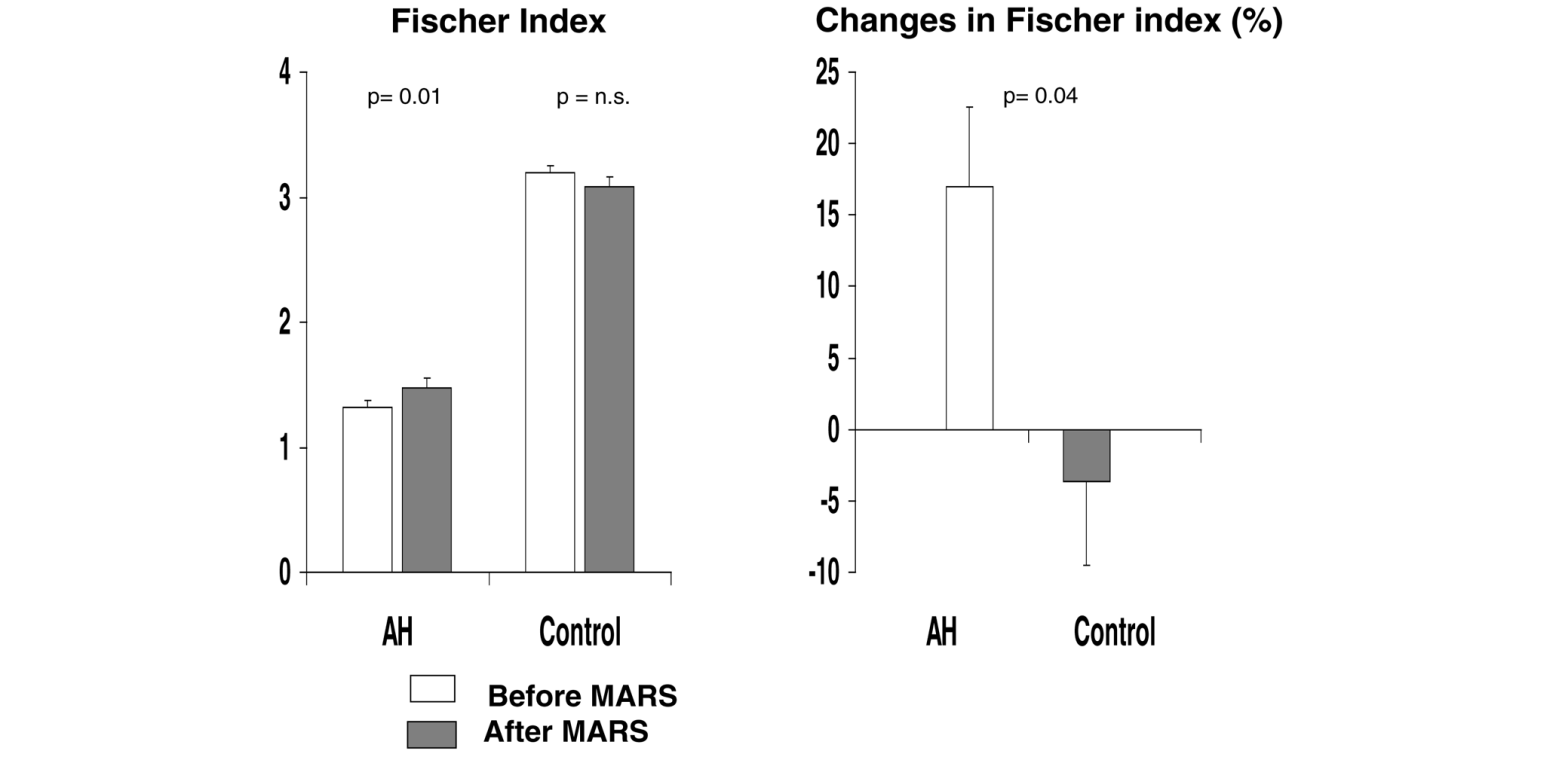
**Changes in the Fischer index**. (Left) Fischer index in patients with alcoholic hepatitis (AH) and pruritus (control) before (empty bars) and after (shaded bars) albumin dialysis. (Right) Percent changes in the Fischer index in patients and controls (filled bar). MARS = Molecular Adsorbent Recirculating System; n.s. = not significant.

**Figure 3:**
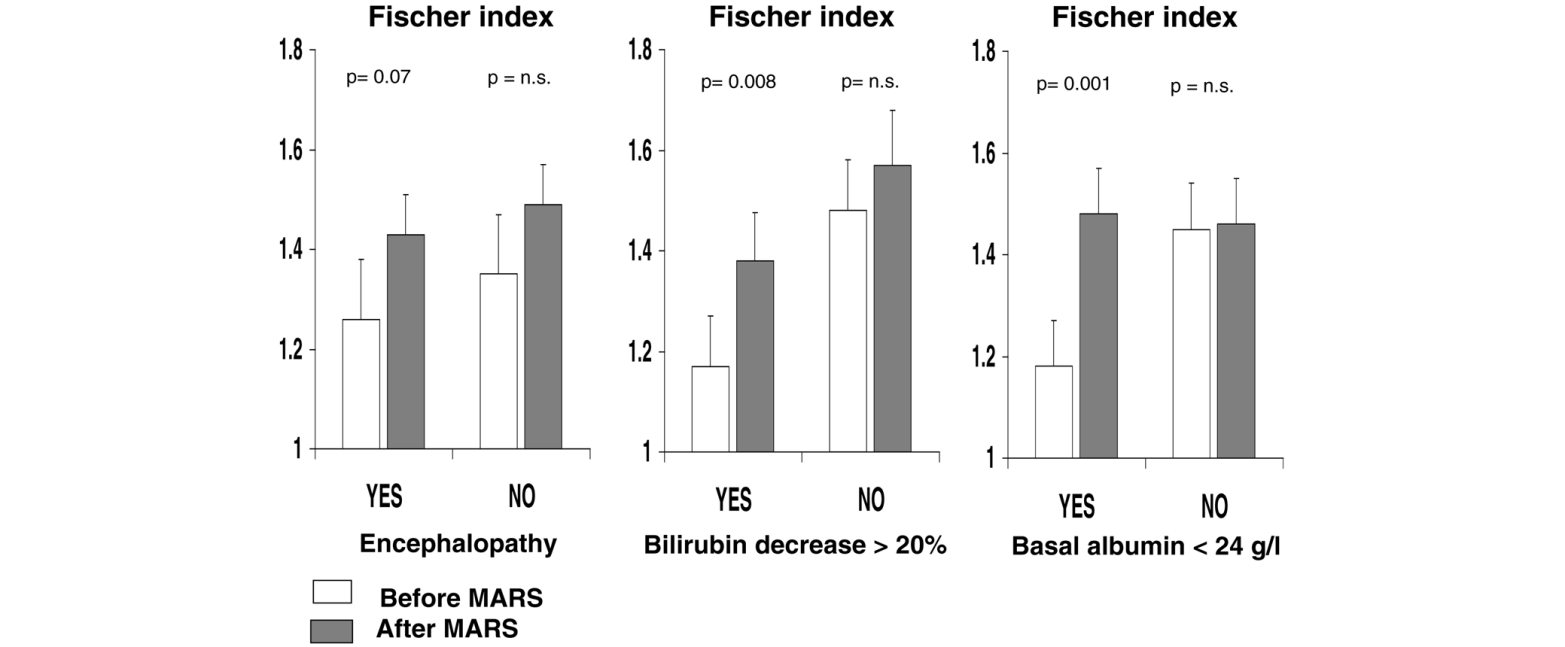
**Fischer index, severity of alcoholic hepatitis and bilirubin decrease after albumin dialysis**. Fischer index in patients with alcoholic hepatitis (AH) and pruritus (control) before (empty bars) and after (shaded bars) albumin dialysis according to (left) the presence of hepatic encephalopathy before treatment, (centre) a bilirubin decrease greater than 20% after albumin dialysis and (right) a baseline serum albumin lower than 24 g/L. MARS = Molecular Adsorbent Recirculating System; n.s. = not significant.

Total amino acid concentrations diminished significantly in patients with alcoholic hepatitis and no changes were observed in the patients with pruritus who served as the control group (Table 2). Circulating branched amino acids were not significantly modified by albumin dialysis, whereas phenolic aromatic amino acids decreased markedly in patients with alcoholic hepatitis and increased in patients with pruritus (Figure 1). Hence, the Fischer ratio increased significantly in patients with alcoholic hepatitis (from 1.32 ± 0.08 to 1.47 ± 0.05; p < 0.01) but decreased in controls (from 3.20 ± 0.16 to 3.08 ± 0.24; p = not significant). Albumin dialysis resulted in a 17% increase in the Fischer ratio in patients with alcoholic hepatitis and a 3% decrease in patients with resistant pruritus (p < 0.05; Figure 2). These changes were more pronounced when corrected by the baseline albumin levels (data not shown). No significant changes were observed in either the circulating levels of tryptophan (from 28 ± 2 μM to 26 ± 1 μM) or ammonia (from 46 ± 6 mM to 41 ± 5 mM). In the alcoholic hepatitis group the changes observed in amino acid levels and the Fischer ratio were more prominent in patients with hepatic encephalopathy before treatment, baseline albumin concentration below 24 g/l, and in those in whom albumin dialysis resulted in a decrease in total bilirubin greater than 20% with respect to pre-treatment levels (Figure 3). The amino acid profile was not significantly modified by albumin dialysis in the group of patients with resistant pruritus.

Six patients with alcoholic hepatitis and the four patients with pruritus were discharged from the hospital after the MARS treatment. The other three patients with alcoholic hepatitis died 16, 36 and 46 days after treatment. The mean hospital stay of patients with alcoholic hepatitis was 21.1 ± 4.1 days (median: 16 days). The three-month, six-month and one-year survival rate of patients with alcoholic hepatitis were 55.5%, 44.4% and 33.3%, respectively. All the patients with pruritus were alive four years after treatment.

## Discussion

Hepatic encephalopathy is a common complication in patients with liver failure, including acute decompensated patients with advanced cirrhosis of different aetiologies, and in patients with severe alcoholic hepatitis. Actually, hepatic encephalopathy is a feature clearly associated with a bad prognosis in patients with this condition [20-23]. The pathophysiology of hepatic encephalopathy is unknown, and different hypotheses have emerged over the years, but portal hypertension and the consequences of liver dysfunction are included in all the proposals, although hepatic encephalopathy may develop into acute liver failure with no significant portal hypertension. There is evidence suggesting that hepatic encephalopathy results from the accumulation of neurotoxic or neuroactive substances in the brain, including ammonia, manganese, aromatic amino acids, mercaptans, phenols, short-chain fatty acids and others.

Prevention and treatment of hepatic encephalopathy relies on the reduction of circulating ammonia either by a reduction in gut production using disaccharides or antibiotics or by increasing its metabolites [24]. There is also some evidence suggesting that increasing the Fischer's ratio may improve hepatic encephalopathy, and this was the basis of the utilisation of branched amino acid supplements in patients with encephalopathy [25-30]. Another way to remove the substances potentially related to hepatic encephalopathy is the utilisation of liver assist devices in order to eliminate or reduce the neurotoxins generated in liver failure.

The results of the current study clearly indicate that albumin dialysis is able to induce favourable effects in patients with severe alcoholic hepatitis. Actually, the degree of hepatic encephalopathy improved in all the cases after albumin dialysis. This positive effect was associated with significant changes in the levels of circulating amino acids, and particularly with changes in phenolic aromatic amino acids, which decreased in patients with alcoholic hepatitis but no changes were observed in the group of patients treated with albumin dialysis for resistant pruritus. As a consequence, the Fischer ratio increased significantly in these patients, but not in the patients with pruritus who had a different baseline amino acid profile. Similar data on the effects of albumin dialysis on the amino acid profile have been reported in patients with acute and acute-on-chronic liver failure [31].

In the current study, the effects of albumin dialysis on Fischer's index were particularly prominent in patients with hepatic encephalopathy, baseline albumin levels below 24 g/l, and in those in which the procedure was apparently most efficient as measured by the decrease in bilirubin levels. Another interesting finding is the fact that the amelioration in the index of hepatic encephalopathy was not related to improvements in variables associated with renal dysfunction, such as creatinine, or the correction in the electrolyte imbalance, therefore, strengthening the effect of albumin dialysis but not of the standard renal dialysis that is able to correct the electrolyte disturbances. In regard to this, recent data indicate that albumin dialysis using the MARS device is an effective approach for improving hepatic encephalopathy in patients with different clinical conditions, but particularly in patients with acute-on-chronic liver failure [13,14,32].

The inability of albumin dialysis to correct ammonia and tryptophan levels is an intriguing finding of this study, thus indicating that improvement in hepatic encephalopathy does not depend exclusively on the normalisation or decrease of these molecules. In this regard, different *in vivo *and *in vitro *studies have suggested that albumin dialysis may improve hepatic encephalopathy by decreasing the circulating levels of both ammonia and tryptophan [33,34]. However, in the current study tryptophan levels were normal in all patients, whereas baseline ammonia was elevated in about 50% of patients. The mechanism by which albumin dialysis decreases phenolic aromatic amino acids in patients with severe alcoholic hepatitis is unknown. However, it could be speculated that the procedure is able to dialyse and adsorb the free circulating levels of these amino acids in patients who have low albumin concentrations, with their serum albumin probably completely saturated and unable to bind or transport more substances. However, further studies must be performed to answer this. The lack of effect of albumin dialysis in significantly modifying the amino acid profile in primary biliary cirrhosis patients with bilirubin, prothrombin index and albumin levels within normal ranges may sustain this explanation. Actually, in these patients albumin dialysis did not induce marked changes in the amino acid profile, but rather a trend to increase the phenolic amino acids after treatment was observed.

On the other hand, it should be taken into account that the effect of albumin dialysis on amino acid profile was more relevant in patients who experienced higher bilirubin decrease after treatment, thus suggesting that the favourable effect on hepatic encephalopathy results from the removal of phenolic amino acids and that the procedure may cause some effect on the removal of other known substances involved in the pathogenesis of encephalopathy. Accordingly, it has been speculated that albumin dialysis is able to remove nitric oxide or a number of cytokines and chemokines probably involved in the pathogenesis of hepatic encephalopathy [35,36]. Thus, the ability of albumin dialysis to remove other toxins and pro-inflammatory stimuli such as lipopolysaccharides and lipid peroxidation end-products may have implications for limiting the inflammatory response that could be implicated not only in renal impairment and circulatory dysfunction but also in the pathophysiology of hepatic encephalopathy in patients with severe liver failure [37]. Recent data support the importance of infection and inflammation even in minimal alteration of cognitive function in patients with liver failure and features of systemic inflammatory response syndrome [37].

## Conclusions

In summary, the results of this study demonstrate that treatment of patients with severe alcoholic hepatitis with albumin dialysis improves hepatic encephalopathy, and that this favourable effect results from the correction of the abnormal amino acid profile, basically by decreasing phenolic aromatic amino acids. These results may explain at least in part the hopeful effects of albumin dialysis on hepatic encephalopathy observed in different trials, although further studies are warranted to define the clinical and biochemical effects of this new procedure in the treatment of patients with acute or acute-on-chronic liver failure.

## Key messages

• Albumin dialysis results in favourable effects in patients with severe alcoholic hepatitis, because hepatic encephalopathy improved in all cases.

• Total amino acid and phenolic aromatic amino acids diminished and the Fischer ratio increased in patients with alcoholic hepatitis treated with MARS.

• Changes in amino acid levels and the Fischer ratio were more prominent in patients with hepatic encephalopathy, low albumin concentration and greater bilirubin extraction with MARS treatment.

• Although the mechanisms by which albumin dialysis decreases phenolic aromatic amino acids in patients with severe alcoholic hepatitis remain unknown, it could be speculated that MARS is able to dialyse and adsorb the free circulating levels of these amino acids in patients who have low albumin concentration, with their serum albumin probably completely saturated and unable to bind or transport more substances.

## Abbreviations

MARS: Molecular Adsorbent Recirculating System; SEM: standard error of the mean.

## Competing interests

The authors declare that they have no competing interests.

## Authors' contributions

AP conceived the study, participated in its design and coordination and performed the statistical analysis. RD participated in the study design and carried out the laboratory analytics. LC, JMS, JC, AE and AM treated the patients, participated in the study design and helped to draft the manuscript. All authors read and approved the final manuscript.
